# Chronic Thromboembolic Pulmonary Hypertension: Treat the Patient Not the Haemodynamics

**DOI:** 10.1155/2012/108672

**Published:** 2012-10-22

**Authors:** Ben Dunne, Annika van den Broek, Vaughan Williams, Gregory Smith, Tamas Revesz, Mark Edwards, Eli Gabbay

**Affiliations:** ^1^Advanced Lung Disease and Transplant Unit, Royal Perth Hospital, Perth, WA 6000, Australia; ^2^Erasmus University, Rotterdam, The Netherlands; ^3^Women's and Children's Hospital, Adelaide, SA 5006, Australia; ^4^Department of Cardiothoracic Surgery, Royal Perth Hospital, Perth, WA 6000, Australia

## Abstract

Chronic thromboembolic pulmonary hypertension (CTEPH) is a disabling condition that is being increasingly recognised. It is unique as a cause of pulmonary hypertension in that it is surgically curable. We wish to highlight the importance of recognition and early referral of any patient who may have CTEPH even in the absence of resting pulmonary hypertension as excellent results can be achieved by restoring pulmonary vascular anatomy, reducing exercise-induced pulmonary hypertension, and reducing dead-space ventilation. We present a case that illustrates these points and discuss our experience as a referral centre for CTEPH.

## 1. Introduction

We report the case of a 15-year-old boy, heterozygous for the antithrombin mutation, who developed chronic thromboembolic pulmonary hypertension (CTEPH) secondary to recurrent pulmonary emboli and was successfully treated with pulmonary thromboendarterectomy.

This case highlights a number of points relevant to both primary care physicians and those working in specialist centres. In particular, we wish to emphasise that significantly symptomatic CTEPH can present with only borderline abnormal resting pulmonary haemodynamics and that it probably remains significantly underdiagnosed. Recognition of this complex condition allows the consideration of curative surgery and a return to normal life for the patient.

## 2. Case Report

Our patient presented at the age of 15 to the ED of an Adelaide hospital with a several-month history of breathlessness, nausea, and dull bilateral chest pain, which worsened over 7 days. He had presented on several occasions in the preceding few months to primary care doctors without an apparent diagnosis. By the time of presentation to the ED, he was noted to be hypoxaemic and had clinical signs of right heart dysfunction (tender hepatomegaly and elevated jugular venous pressure) without dependent oedema.

He was known to have antithrombin deficiency due to a heterozygous mutation, being diagnosed at the age of 7 after screening in view of a strong family history on his maternal side. His AT III levels were recorded at 38% predicted, which is lower than the generally reported range of 40–60% associated with heterozygosity [1]. He had no other known risk factors for venous thromboembolism.

CTPA demonstrated evidence of acute bilateral submassive pulmonary emboli extending from both left and right pulmonary arteries to the subsegmental level involving all lobes of the lungs. Further, there was evidence of chronic thromboembolism with webs and cutoffs in both segmental and subsegmental arteries suggesting previous pulmonary thromboembolic events.

Despite therapeutic anticoagulation, and an initial improvement, he failed to progressively improve over the following 6 months. He remained breathless on minimal exertion, was unable to attend school, and had difficulty performing activities of daily living due to intense nausea and breathlessness.

 A 6-minute walk test was attempted but abandoned as he desaturated to 80% on air after just 20 meters accompanied by severe dyspnoea.

A V/Q scan demonstrated evidence of incomplete improvement in pulmonary clot burden with bilateral asymmetric mismatched defects worse on the left. Echocardiography revealed a right ventricular systolic pressure (RVSP) of 45 mmHg.

As pulmonary endarterectomy is not offered in South Australia, he was referred to Royal Perth Hospital for consideration. Repeat CTPA as well as formal pulmonary angiography revealed evidence of residual left worse than right upper and lower lobar arterial clot. Echocardiography confirmed the mildly elevated RVSP. 

**Figure 1:**
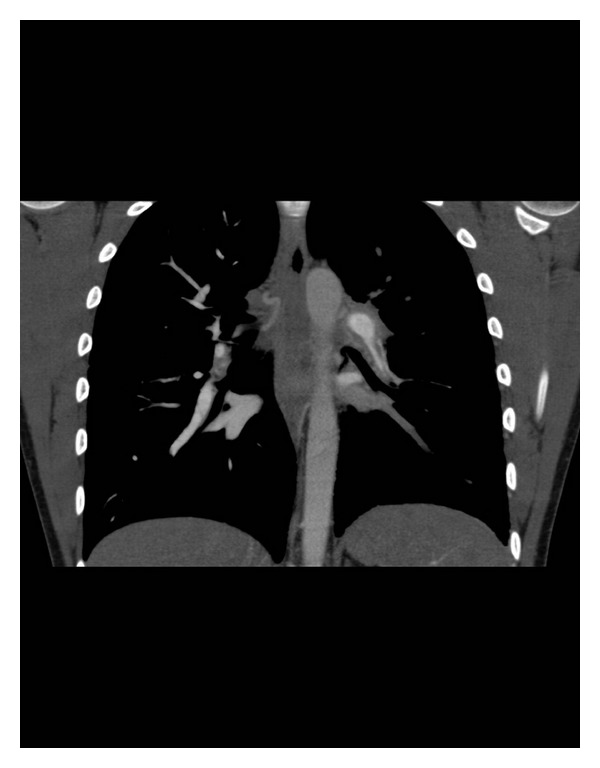
Coronal CT Image Demonstrating Pulmonary Arterial Thromboembolic Disease.

Right heart catheterisation was performed confirming mild resting precapillary pulmonary hypertension (Figure 1 and Table 1).

In view of the (only) mild elevation in pulmonary vascular resistance, which appeared out of keeping with the degree of symptomatic impairment, exercise pulmonary haemodynamics were attempted but unfortunately, the patient became extremely unwell on only minimal exertion and before stable reliable exercise haemodynamics could be recorded, and the procedure was abandoned.

After discussion at the Royal Perth Hospital Advanced Lung Disease and Pulmonary Vascular Unit Multidisciplinary meeting, a consensus was reached to proceed with pulmonary endarterectomy. The decision was made due to our view that the patient's marked symptomatology and exercise desaturation reflected a significant worsening of cardiopulmonary haemodynamics and dead space ventilation with exercise.

Pulmonary endarterectomy was carried out in the standard fashion through a median sternotomy, with cardiopulmonary bypass and circulatory arrest. The left upper lobe was successfully endarterectomised, however, the left lower lobe was only partially endarterectomised as the disease was very distal. Unfortunately there was no surgically resectable disease in the right pulmonary arterial tree.

He recovered well, with a postoperative ICU stay of 18 hours and a total post-operative stay of 6 days without complication.

His anticoagulation was managed perioperatively with daily IV antithrombin replacement (Thrombotrol) and intravenous heparin until he was reestablished on warfarin with a target INR of 3–3.5. 

Six months postoperatively he is well. He has been able to return to school as well as normal activities and sports. His six minute walk test is normal (predicted for age) at 690 metres and exercise oximetry has shown no desaturation even after strenuous exercise. He continues on warfarin with a target INR of 3–3.5. 

## 3. Discussion

Chronic thromboembolic pulmonary hypertension (CTEPH) is an uncommon condition that is being increasingly recognised. Once thought to occur in less than 1% of patients who suffer from an acute pulmonary embolus, its incidence is now estimated as at least 3.8% [2] and is still probably underdiagnosed. It is unique as a cause of pulmonary hypertension as it is amenable to complete cure with a surgical procedure other than lung transplantation. Pulmonary endarterectomy affords these patients the opportunity to return to a normal level of physical functioning and quality of life [3]. 

This case illustrates several issues. Significant symptoms especially on exercise may develop in patients with CTEPH even in the absence of widespread clot burden. We have previously reported our experience in measuring exercise haemodynamics in patients with mild or borderline resting elevation in pulmonary vascular resistance [4]. Performing exercise haemodynamics, especially in patients with symptomatology that appears “out of keeping” from resting pulmonary haemodynamics can “uncover” significant abnormalities with exercise. This may be especially relevant to patients with CTEPH [5].

 It is our view that the presence of exercise-induced pulmonary hypertension resulting from chronic thromboembolic disease may be an indication for surgery even in the absence of abnormal resting haemodynamics provided the patient is sufficiently symptomatic and where there is sufficient surgically accessible clot burden.

There is evidence that without surgery, the degree of pulmonary hypertension will worsen with time [6] and that the same functional outcome can be achieved for the patient without waiting for resting pulmonary hypertension to develop. Further, there is a lower operative mortality rate in patients with lower resting pulmonary vascular resistance (PVR) [5].

This case also highlights the importance of fully investigating unexplained breathlessness in a young patient, which in our experience is too often dismissed. This is of particular importance in the setting of a family history of thrombophilia, PAH, or cardiac disease. Pulmonary hypertension of any cause can often present with little abnormalities on examination or on basic investigation and CTEPH in particular can often present in the absence of a history of venous thromboembolism [6]. 

A common but underappreciated presentation of pulmonary hypertension in young patients is nausea and early satiety caused by right heart failure and gastric congestion. This is often present before the more traditionally described physical signs of right heart failure are apparent. 

Most importantly, any patient suspected of potentially having CTEPH should be referred to a specialist centre with significant experience in its management. The decision to proceed with endarterectomy is complex and involves consideration of symptoms, the presence and burden of apparent surgically resectable disease, and the presence of abnormal pulmonary haemodynamics at rest and/or on exercise.

We believe that the symptomatic improvement in the subgroup of patients with mildly raised resting pulmonary pressures is often due not only to the reduction in pulmonary vascular resistance on exertion but to restoration of normal (or near-normal) pulmonary vascular anatomy and reduced dead-space ventilation as evidenced by the improved exercise oximetry in this patient. 

In conclusion, we believe that this case not only highlights the importance of earlier recognition of CTEPH as well as other causes of pulmonary hypertension but also the importance of referral to a specialist centre experienced in the assessment and surgical management of this potentially curative disease. The decision to proceed with surgery involves consideration of the patient's symptoms and pulmonary arterial anatomy and not only the haemodynamics.

## Figures and Tables

**Table 1:** Right heart catheter results.

Cardiac index	3.5
Mean PA pressure	26 mmHg
Pulmonary artery wedge pressure	10 mmHg
PVR	2.46 Woods units
PVRI	365
